# Early detection of pediatric health risks using maternal and child health data

**DOI:** 10.1038/s41598-024-65449-8

**Published:** 2024-07-04

**Authors:** Cornelia Ilin

**Affiliations:** University of California, Berkeley, CA USA

**Keywords:** Diseases, Diagnosis

## Abstract

Machine learning (ML)-driven diagnosis systems are particularly relevant in pediatrics given the well-documented impact of early-life health conditions on later-life outcomes. Yet, early identification of diseases and their subsequent impact on length of hospital stay for this age group has so far remained uncharacterized, likely because access to relevant health data is severely limited. Thanks to a confidential data use agreement with the California Department of Health Care Access and Information, we introduce Ped-BERT: a state-of-the-art deep learning model that accurately predicts the likelihood of 100+ conditions and the length of stay in a pediatric patient’s next medical visit. We link mother-specific pre- and postnatal period health information to pediatric patient hospital discharge and emergency room visits. Our data set comprises 513.9K mother–baby pairs and contains medical diagnosis codes, length of stay, as well as temporal and spatial pediatric patient characteristics, such as age and residency zip code at the time of visit. Following the popular bidirectional encoder representations from the transformers (BERT) approach, we pre-train Ped-BERT via the masked language modeling objective to learn embedding features for the diagnosis codes contained in our data. We then continue to fine-tune our model to accurately predict primary diagnosis outcomes and length of stay for a pediatric patient’s next visit, given the history of previous visits and, optionally, the mother’s pre- and postnatal health information. We find that Ped-BERT generally outperforms contemporary and state-of-the-art classifiers when trained with minimum features. We also find that incorporating mother health attributes leads to significant improvements in model performance overall and across all patient subgroups in our data. Our most successful Ped-BERT model configuration achieves an area under the receiver operator curve (ROC AUC) of 0.927 and an average precision score (APS) of 0.408 for the diagnosis prediction task, and a ROC AUC of 0.855 and APS of 0.815 for the length of hospital stay task. Further, we examine Ped-BERT’s fairness by determining whether prediction errors are evenly distributed across various subgroups of mother–baby demographics and health characteristics, or if certain subgroups exhibit a higher susceptibility to prediction errors.

## Introduction

Early identification of diseases and their associated length of hospital stay (LoS) is vital for better treatment options, more effective follow-up arrangements, longer survival rates, improved long-term outcomes, and lower hospital utilization costs.

In recent years, breakthrough progress in diagnosis prediction was made by leveraging electronic health records (EHR) and advanced deep learning (DL) architectures, such as convolutional neural networks (CNN, e.g., Nguyen et al. (Deepr)^1^), recurrent neural networks (RNN, e.g., Choi et al. (Doctor AI)^2^), long short-term memory networks (LSTM, e.g., Pham et al. (DeepCare)^3^), and an even more powerful architecture called Bidirectional Encoder Representation from Transformers (BERT). For instance, Li et al.^4^ introduce BEHRT, a BERT-inspired model applied to EHR, capable of predicting the likelihood of more than 300 conditions in one’s future medical visit; Shang et al.^5^ propose G-BERT, a model that combines the power of graph neural networks (GNN) and BERT for diagnosis prediction and medication recommendation; Rasmy et al.^6^ introduce Med-BERT, also a BERT model, to provide pre-trained contextualized embeddings run on large-scale structured EHR. However, a very limited number of studies focus on leveraging EHR and state-of-the-art DL architectures for the task of predicting hospital LoS^7,8^. For instance, Song et al.^7^ develop SAnD (Simply Attend and Diagnose), a DL-inspired model, to predict diagnosis codes and LoS, among other tasks, using a multi-class classification approach. Their LoS estimation is based on analyzing events occurring hourly from admission time. Additionally, Hansen et al.^8^ introduce M-BERT, a BERT-inspired model applied to sequences of patient events gathered within the first 24 h of admission for binary, multi-class, and continuous LoS prediction.

To the best of our knowledge, most advances in this literature (a) rely on EHR representative of the adult population^4,7,9^; (b) need to specify the patient age distribution^1,2,5,6,8,10–13^; (c) estimate how long a patient is likely to stay in the hospital after being admitted, however, forecasting LoS before admission is equally pertinent in preventive healthcare and optimizing hospital resource allocation^7,8^; (d) use models that focus on predicting diagnosis or LoS for a limited set of health outcomes^3,10,14^; (e) focus on improving health risk assessment performance by accounting only for the timing irregularity between clinical events (e.g., age at the time of visit)^1,2,4,8^; (f) do not report prediction performance on rare diseases^15^, or (g) do not use in-utero health information for diagnosis prediction.

However, computer-aided early detection of diseases and their associated LoS holds particular significance in the field of pediatrics. Timely diagnosis and intervention are crucial for enhancing the long-term well-being of children, as highlighted in various studies^14–18^. Consequently, we develop Ped-BERT, an architecture inspired by BERT^19^. Our model accurately predicts over 100 potential primary diagnoses and the length of hospital stay that a child might face during their upcoming medical visit, by relying on pre-trained diagnosis embeddings. We evaluate our approach against two contemporary classifiers (a logistic regression and a random forest) and two state-of-the-art DL classifiers (a pre-trained transformer decoder and a neural network with randomly initialized embeddings). Thus, our analysis could serve as a valuable tool for assisting researchers in utilizing machine learning for pediatric healthcare guidance, therefore aiding pediatricians in their clinical decision-making processes.

Ped-BERT leverages a rich dataset encompassing hospital discharge records and emergency room information for pediatrics, including the patient’s age and the residential zip code or county at the time of the visit. Additionally, it can optionally integrate maternal health data from both pre- and postnatal periods. To the best of our knowledge, our prediction framework, leveraging data that matches mother and baby pairs longitudinally is the first of its kind. Furthermore, this dataset empowers us to explore the model’s capability to simultaneously predict primary diagnosis and LoS in the next medical visit, and to assess its overall fairness, including an examination of whether prediction errors are evenly distributed across different demographics of mother–baby pairs.

To summarize, we contribute to the literature as follows: first, we use a novel data set that links medical records of mother–baby pairs between 1991 and 2017 in California; second, we develop Ped-BERT, a DL architecture for early detection prediction of health risks for pediatric patients seeking care in inpatient or emergency settings, and compare its performance against other contemporary or state-of-the-art classifiers; third, we leverage both temporal and spatial patient characteristics, such as age and geographical location at the time of visit; fourth, we assess improvements in model performance when incorporating mother’s attributes data, such as the mother’s pre- and post-partum health history, and fifth, we evaluate Ped-BERT’s performance with fairness in mind.

## Data

This study relies on data from the California Department of Health Care Access and Information (HCAI^20^). Through a confidential data use agreement, we access the universe of births between 1991 and 2012 (Birth data), patient discharge data (PDD), and emergency department visits (EDD) through 2017 from nearly 7000 California licensed healthcare facilities^21^. We use this data to pre-train and fine-tune Ped-BERT.

### Birth data

**Figure 1:**
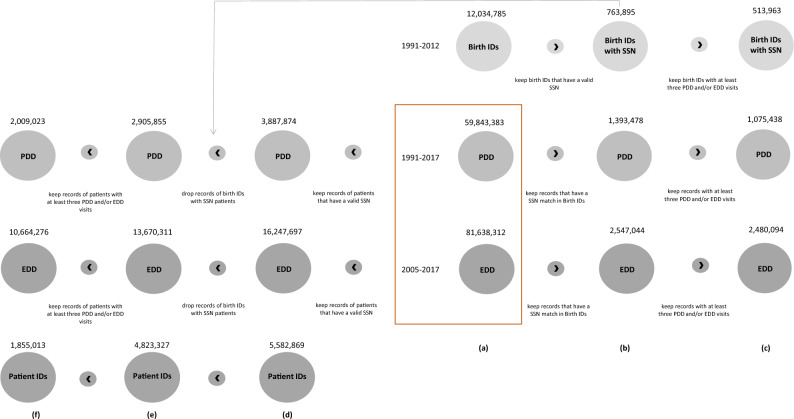
Filtering, linking, and summary of our data. (**a**, **b**) From the initial set of 12M birth IDs, 59.8M patient discharge data records (PDD), and 81.6M emergency department data records (EDD), we only retain those that can be linked via SSN at birth or in the first year of life: 764K, 1.4M and 2.5M, respectively. (**c**) We further filter by number of inpatient/emergency encounters, only retaining records for patients with at least three medical encounters. This final set consists of approximately 3.5M hospital visits (PDD and EDD combined) between 1991 and 2017 for 513, 963 mother–baby pairs. This data is used for fine-tuning Ped-BERT. (**d**) From the initial set of 59.8M PDD records and 81.6M EDD records, we only retrain those that can be linked via SSN at some point in life: 2.9M and 13.6M, respectively. (**e**,**f**) We further drop the records of patients whose SSN has a match in the 513, 963 mother–baby pairs data or have less than three inpatient/emergency encounters. This final set consists of around 2M and 10M records in the PDD and EDD data, respectively, corresponding to 1, 855, 013 unique patient IDs. We use this data for pre-training Ped-BERT.

We observe over 12M birth records registered in California, including maternal antepartum and postpartum hospital records for the 9 months before delivery and 1-year post-delivery (Fig. 1a, top panel). We filter the data to retain only mother–baby pairs (birth IDs) for which the discharge records link to birth certificate data and the baby’s social security number (SSN), if the SSN was assigned either at birth or within their first year of life. After filtering, our birth data includes 763,895 mother–baby pairs whose medical records can be tracked over time by linkage with the PDD and EDD data via the SSN (Fig. 1b, top panel). Among all variables present in the birth data, we retain information on the baby’s gender, race, and residency zip code and county at birth. We also include information on the mother’s race and education, the month prenatal care began, the number of prenatal visits, and the number of times the mother visited a healthcare facility in an emergency or inpatient setting 9 months before and 12 months after birth.

### Patient discharge and emergency department visits

The PDD and EDD datasets consist of over 59M inpatient discharges between 1991 and 2017 and over 81M in emergency visits between 2005 and 2017, respectively (Fig. 1a, middle and bottom panels). If the emergency encounter resulted in a same-hospital admission, the record reflects the inpatient encounter, and no separate emergency department visit is recorded.

We subset these data to include only those records for which the patient’s SSN has a match in the Birth data (Fig. 1b, middle and bottom panels). To improve our machine learning task, we further filter this data to select only those patients whose medical history includes at least three emergency or inpatient stays. After this last filtering, we have nearly 1M inpatient and 2.5M emergency discharge records for 513,963 mother–baby pairs (Fig. 1c, middle, bottom, and top panels). From the PDD and EDD data, we retain information on patient demographic characteristics (including residence zip code and county at the time of visit), visit LoS, and up to three disease codes as listed by the healthcare provider during the encounter. The LoS variable in our data is derived by computing the difference between the discharge and admission dates. For instance, if a patient is admitted on one day and discharged the next, their LoS is recorded as one day. Patients admitted and discharged on the same day are assigned a LoS of zero days. Similarly, emergency room patients who are not transferred to an inpatient setting are also recorded with a LoS of zero days. The disease codes in our data are classified using the 9th and 10th revisions of the International Statistical Classification of Diseases and Related Health Problems (ICD-9 and ICD-10, respectively). For ease of analysis and interpretability, we convert ICD-10 to ICD-9 codes using the AtlasCUMC dataset^22,23^ and choose to operate at the two-digit sub-chapter level.

Via a random split, we use 70%, 10%, and 20% of these 513, 963 mother–baby pairs, respectively, for fine-tuning Ped-BERT and for assessing prediction performance in the downstream tasks of predicting the next principal medical diagnosis and associated hospital LoS. In the following, we refer to these three data sets simply as ‘fine-tuning training set’, ‘fine-tuning validation set’, and ‘fine-tuning test set’.

### Ped-BERT pre-training data

For the pre-training of Ped-BERT, it is important to highlight that our goal is to utilize patient records without matches in the fine-tuning data but with available SSN information that enables us to establish connections across time. This distinction is crucial because the data used for pre-training Ped-BERT should not align with our final prediction task to prevent data leakage.

We begin with the raw dataset comprising over 59M inpatient discharges (PDD data) and over 81M emergency visits (EDD data) (Fig. 1a, middle and bottom panels). From this extensive dataset, we retain records of patients with valid SSN. Following this filtering process, we are left with nearly 3.8M inpatient stays and 16.2M emergency visits, corresponding to nearly 5.5M patient IDs (Fig. 1d). Subsequently, we exclude all patients whose SSN match the 513, 963 birth IDs described in the previous subsection because we will use this data for fine-tuning Ped-BERT (Fig. 1e). Finally, to improve our machine learning task, we further refine the data to include only patients with a minimum of three medical encounters. This step leaves us with approximately 2M inpatient discharges and 10M in emergency room visits, totaling 1,855,013 unique patients for pre-training Ped-BERT (Fig. 1f).

Via a random split, we use 80% and 20% of these data, respectively, for pre-training Ped-BERT and validating prediction performance. In the following, we refer to these two data sets simply as ‘pre-training training set’ and ‘pre-training validation set’.

### Patient medical history

**Figure 2:**
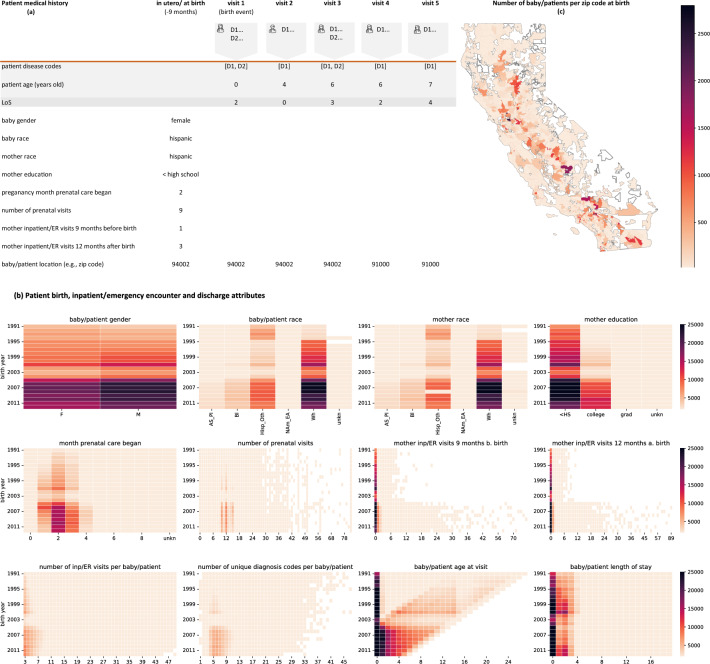
Patient medical history and descriptive statistics. (**a**) Example, in tabular form, of a patient’s medical history documenting data collected in the in-utero period or at the time of birth, and during the patient’s first five inpatient/emergency visits. (**b**,**c**) Summary statistics for mother–baby/patient demographics and health-related outcomes belonging to the 513, 963 mother-baby pairs used for fine-tuning Ped-BERT. (**c**) The map was generated using the geopandas and matplotlib libraries available in Python. *F* Female, *M* Male, *AS_PI* Assian_Pacific Islander, *Bl* Black, *Hisp_Oth* Hispanic_Other, *NAm_EA* Native American_Eskimo_Aleut, *Wh* White, < *HS* less than High-school, *grad* graduate education, *b.* before, *a.* after, *unkn* unknown.

For our fine-tuning task of predicting the principal diagnosis and LoS in the upcoming medical visit, we rely on patient health information, starting nine months before birth, until data censoring. Let **P** represent our sample of patients, and **T** represent a set of sorted time stamps. In our data, each patient $p \in \{1, 2, \ldots , P\}$, is described by a set of birth and mother attributes, $p.A_{bm}=\{A_1,A_2,\ldots ,A_n\}$ recorded in the prenatal period and/or at the time of birth and in the mother’s pre- and postnatal period. Each patient is also characterized by a set of inpatient/emergency encounter attributes, $p.A_{ie}=\{(A_1,A_2,\ldots ,A_n|1), (A_1,A_2,\ldots ,A_n|2),\ldots ,(A_1,A_2,\ldots ,A_n|T)\}$ recorded at time $t \in \{1, 2, \ldots T\}$ of encounter with the medical provider. Separately, we denote each patient’s LoS attribute by $p.A_{d}=\{(A_{LoS}|1), (A_{LoS}|2), \ldots ,(A_{LoS}|T)\}$ recorded at time of discharge. The attributes in $p.A_{bm}$ cover the baby’s gender and race, mother’s race and education, pregnancy month prenatal care began, the number of prenatal visits, mother inpatient/emergency visits nine months before and twelve months after birth delivery, and residency zip code/county at birth. Similarly, the attributes in $p.A_{ie}$ are sequences of patient disease codes, patient age, and patient residency zip code/county at the time of visit. Figure 2a illustrates, in tabular form, the medical history of a hypothetical patient with birth and mother attributes (data column 2) $p.A_{bm}=\{$female, hisp, hisp, < high school, 2, 9, 1, 3, 94002$\}$, medical encounter attributes (data columns 3–7) $p.A_{ie}=\{$([D1, D2], 0, 94002 | visit = 1), ([D1], 4, 94002 | visit = 2), ..., ([D1], 7, 91000 | visit = 5)$\}$, and LoS discharge attribute $p.A_{d}=\{$ (2 | visit = 1), (0 | visit = 2), ..., (4 | visit = 5)$\}$. The diagnosis codes assigned by medical personnel are represented as D1, D2, ...etc.

Descriptive statistics of the data utilized for pre-training Ped-BERT can be found in Supplementary Fig. S1, and those for fine-tuning Ped-BERT are presented in Fig. 2. In terms of birth and mother attributes, the distribution of the baby/patient’s race is approximately even between males and females; both the baby/patient’s and mother’s race are predominantly white or Hispanic/other; most mothers have attained an educational level below high school or have completed college; prenatal care typically starts within 1–3 months of conception, with most mothers receiving 10–12 prenatal care visits; a majority of mothers in our data did not require inpatient or emergency room services in the prepartum and postpartum period. Regarding baby/patient healthcare utilization, the majority of subjects in our dataset have between 3–7 inpatient/emergency visits, with a LoS less than or equal to 3 days, and between 5–9 unique diagnosis codes (see Fig. 2b for additional insights). Finally, mother–baby pairs in our dataset are evenly distributed across California (Fig. 2c).

## Methods

This study aims to introduce Ped-BERT, a BERT transformer-encoder-based architecture^19,24^. Ped-BERT consists of a bidirectional training procedure and masked language modeling approach (MLM), which enable the model to learn the probability distribution of different health outcomes in a pediatric patient’s next medical visit. We describe our methodology below.

### Models

We decompose our prediction task into two components. In the first step, we pre-train our Ped-BERT model using each patient’s inpatient/emergency encounter attributes data, $p.A_{ie}$, and BERT’s MLM approach. The objective here is to learn good disease representations. Afterward, via the second step, we fine-tune Ped-BERT’s parameters in a supervised fashion via the downstream task of predicting the principal diagnosis and the corresponding LoS in the next visit. In adherence to regulations concerning protected data, all procedures were conducted following relevant guidelines and regulations. Experimental protocols received approval from both the California Committee for the Protection of Human Subjects (CPHS) and the Institutional Review Board (IRB) at the University of Wisconsin Madison. Informed consent was secured from all subjects or their legal guardians, as documented per CPHS data request protocols.

### Ped-BERT pre-training

The pre-training stage is concerned with learning good disease embeddings. Concretely, Ped-BERT pretrains bidirectional diagnosis representations from medical histories by jointly conditioning both left and right diseases in a pediatric patient’s medical history. This approach has been shown to outperform other deep learning architectures, such as CNN, RNN, and LSTM^1–3,9^, or left-to-right attention as presented in the original transformer architecture^24^. In addition, Ped-BERT is pre-trained using the MLM approach, whose objective is to randomly replace a fraction of the diagnosis codes with mask tokens [MASK] and task the model with predicting these hidden disease codes instead.

**Figure 3:**
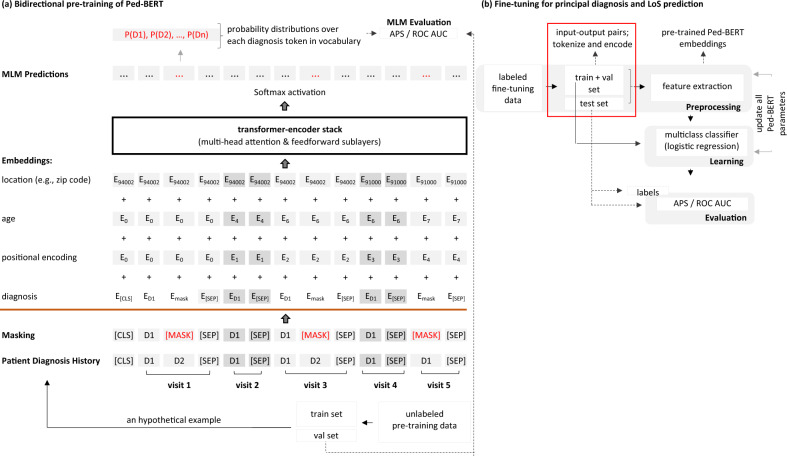
Ped-BERT architecture. (**a**) The pre-training task is explained using as an example the hypothetical patient introduced in Fig. 2a: Ped-BERT sees the medical history and masks some of the diagnosis codes before sending them to embedding, multi-head attention, and feedforward network sublayers. The task here is to predict the [MASK] disease codes. (**b**) In the fine-tuning task, the pre-trained Ped-BERT model parameters are fine-tuned using a NN with one hidden layer with the objective of predicting the probability distribution over given diagnosis codes and the LoS in a pediatric patient’s next visit. The fine-tuning and pre-training steps are evaluated using the APS and ROC AUC scores.

This stage relies on the unlabeled pre-training data split into ‘pre-training training set’ and ‘pre-training validation set’, and for simplicity, Fig. 3a illustrates the pre-training task of Ped-BERT using as an example the hypothetical patient introduced earlier (see Fig. 2a). First, the model is given the patient’s health history in the following format: **[CLS]** $D_1$ $D_2$ **[SEP]** $D_1$ **[SEP]** $D_1$ $D_2$ **[SEP]** $D_1$ **[SEP]** $D_1$ **[SEP]**. Here, [CLS] is a token denoting the beginning of the patient’s medical history, and the [SEP] token is added to indicate the end of a medical visit. Both tokens, [CLS] and [SEP], are added to aid with the subsequent diagnosis prediction task. The *D* tokens represent up to three medical diagnoses at the time of visit (see Fig. 3a—Patient Diagnosis History)

Second, the data undergoes pre-processing for the MLM task, involving the random selection of 15 percent of the disease tokens for masking (see Fig. 3a—Masking). The selection/masking process follows the original BERT model^19^.

Third, a trainable input embedding matrix is created. We first identify the unique diagnosis codes in the masked training data, map them to integer values, and then encode each patient’s diagnosis history using this mapping. Since our disease sequences have different lengths, we use zero padding as a placeholder for adjusting sequence length. We continue by encoding information on visit position, patient’s age, and geographical location to give our model a sense of the timing, age, and location of events. While age embeddings have been used before (e.g., BEHRT^4^), the geographical location is unique to Ped-BERT. We hypothesize that one’s location could be an essential determinant of health outcomes due to the environmental impacts of the quality of local resources, such as clean air and safe water, for example. These resources are prerequisites for health, and poor attributes can be particularly detrimental to vulnerable populations such as the very young. We pre-train Ped-BERT using the ‘pre-training training set’ with different input embedding specifications. We define our baseline specification as the sum of diagnosis and positional embeddings. We then assess for any MLM prediction performance improvement by adding age and location embeddings (see Fig. 3a— Embeddings).

Finally, the output of the input embeddings sublayer is sent to multi-head attention and feedforward network sublayers (see Fig. 3a—transformer-encoder stack). The multi-head attention sublayer is followed by post-layer dropout and normalization. The output is passed to the fully connected feedforward network sublayer and followed by post-layer normalization. This last layer produces the logits for each token in the diagnosis vocabulary. The predicted masked token is extracted from these logits using a softmax activation function, which provides a probability distribution over each diagnosis token in the vocabulary (see Fig. 3a—MLM Predictions). We keep the ‘pre-training validation set’ for model validation and evaluation (see Fig. 3a—Evaluation).

### Ped-BERT fine-tuning for diagnosis and LoS prediction

A complete training procedure of Ped-BERT includes fine-tuning the model for specific downstream tasks using labeled data. Concretely, our main objective in the fine-tuning stage is to predict the probability distribution over a set of principal diagnosis codes and associated hospital LoS in a pediatric patient’s next inpatient or emergency room visit. Regarding diagnosis, we choose to focus on predicting the principal code only because, according to HCAI’s Patient Discharge Data (PDD) dictionary^25^, the principal diagnosis is defined as “the condition established, after study, to be the chief cause of the admission of the patient to the hospital for care”. Figure 3b shows the workflow for applying the pre-trained Ped-BERT to these two predictive tasks.

We start from the labeled fine-tuning data split into ‘fine-tuning training set’, ‘fine-tuning validation set’, and ‘fine-tuning test set’. For each patient *p* and each data partition, we randomly choose a visit index *v* ($2 \le v < T$) to split their health attributes data, $p.A_{ie}$ into input-output pairs. The input is denoted by $X_{p.A_{ie}}=\{(A_\text {disease codes}, A_\text {age}, A_\text {location}|1), \ldots , (A_\text {disease codes}, A_\text {age}, A_\text {location}|v)\}$. For the diagnosis prediction task, the output $y^d_{p.A_{ie}}$, is a multi-hot vector of length 115 (corresponding to the total number of disease codes in Ped-BERT’s vocabulary) equal to 1 for the principal diagnosis code that exists in the next visit, $A_\text {principal disease code}|v+1$. For the LoS task, the output, $y^{LoS}_{p.A_{ie}}$, is a multi-hot vector of length 3, equal to 1 if the LoS in the next visit, $A_\text {LoS}|v+1$, corresponds to one of these three categories: LoS $\ge 1$ day, 1 day > LoS $\ge 3$ days, LoS $>3$ days.

We tokenize and encode the diagnosis history (and optionally, age and location history of each patient), and feed the data into Ped-BERT for embeddings extraction (based on the output of the last layer of the transformer-encoder block, see Fig. 3b—Preprocessing). We then use the ‘fine-tuning training set’ to update all Ped-BERT’s learned parameters by fitting and optimizing a multiclass NN with one hidden layer for subsequent primary diagnosis and LoS prediction (see Fig. 3b—Learning). We use the ‘fine-tuning validation set’ for hyper-parameter tuning, and keep the ‘fine-tuning test set’ until the very end for the final model evaluation (see Fig. 3b—Evaluation).

### Prediction performance evaluation

We evaluate the performance of both the pre-trained and fine-tuned Ped-BERT model using two key metrics: the Average Precision Score (APS) and the Area Under the Receiver Operating Curve (ROC AUC). Note that the APS summarizes a precision-recall curve as the weighted mean of precisions achieved at each threshold, with the increase in recall from the previous threshold used as the weight (see scikit-learn^26^ for implementation details).

During the pre-training phase, we calculate these metrics by comparing the model’s predictions to the actual ground-truth data associated with the [MASK] token for all patients within the “pre-training validation set”. In the fine-tuning stage, we represent the model’s predictions for each patient *p* as {$y^{*d}_{p.A_{ie}}$, $y^{*LoS}_{p.A_{ie}}$} and gauge the model’s performance by assessing the agreement between these predictions ({$y^{*d}_{p.A_{ie}}$, $y^{*LoS}_{p.A_{ie}}$}) and the actual values ({$y^d_{p.A_{ie}}$, $y^{LoS}_{p.A_{ie}}$}). This assessment is conducted by computing the APS and ROC AUC on the “fine-tuning test set” individually for each patient, and subsequently calculating the averages across all patients for all diagnosis and LoS classes, as well as the averages across all patients for each specific diagnosis and LoS class.

## Results

We present results from Ped-BERT’s pre-training stage and then evaluate Ped-BERT’s fine-tuned prediction ability for our two downstream tasks. We conclude by discussing the results of a few fairness tasks and how Ped-BERT could guide researchers and medical practitioners.

### Ped-BERT pre-training evaluation

The Ped-BERT architecture is determined by computational constraints and performance evaluation on the ‘pre-training validation set’, featuring the following specifications: the input diagnosis embedding matrix is of size 120 $\times$ 128, with the first dimension representing the length of the diagnosis vocabulary (115 unique two-digit diagnosis codes + *OOV* + [*MASK*] + [*CLS*] + [*SEP*] + padding token) and the second dimension representing the embedding size; the patient history is restricted to a maximum length of 40 tokens; the encoder is a stack of 6 identical layers; inside each of these identical layers there is a multi-head attention sublayer containing 12 heads and a feedforward network sublayer containing 128 hidden units; the dropout regularization rate is set to 0.1; pre-training is for 15 epochs using the Adam optimizer with a learning rate of $3e-5$ and a decay of 0.01. More details on hyperparameter search can be found in Supplementary Table S1.

**Figure 4:**
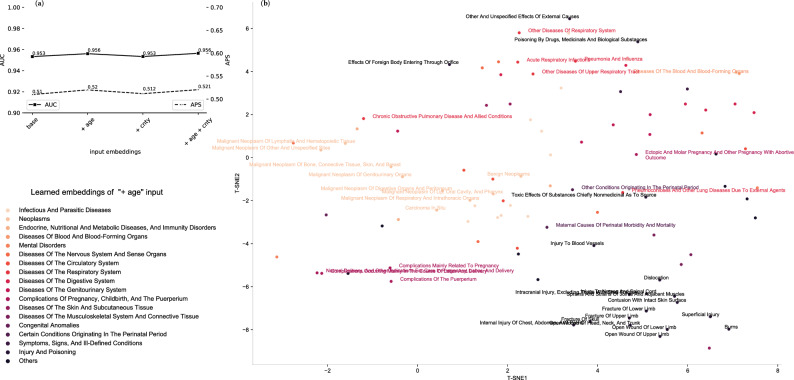
Evaluation of Ped-BERT’s MLM task. (**a**) The average precision score (APS, right y-axis) and the area under the receiver operating curve (ROC AUC, left y-axis) are computed as sample averages for the following embedding specifications: base (which is the sum of diagnosis embeddings and positional encodings), base + age, base + county, and base + age + county embeddings. These metrics represent comparisons between the ground truth (unmasked tokens) and the MLM-predicted diagnosis (masked tokens) in the ‘pre-training validation set’. (**b**) Intrinsic evaluation of the MLM embeddings via visual inspection for the base + age input embeddings specification. We reduce the dimension of the embedding matrix from $120 \times 128$ to $120 \times 2$ using t-SNE to create a 2D visualization of all 115 two-digit diagnosis codes in our vocabulary. Colors represent ICD9 diagnosis chapters.

Ped-BERT is pre-trained using different specifications for the input embedding matrix. As mentioned in the “Methods” section, we define our baseline embeddings specification as the sum of diagnosis embeddings and positional encodings. We then augment this baseline by adding age embeddings (+ age), county embeddings (+ cnty), and age + county embeddings (+ age + cnty). Figure 4a presents a couple of interesting findings: adding age embeddings improves the APS score relative to baseline [0.52 vs. 0.51]; adding county embeddings to the baseline + age specification results in negligible APS differences [APS: 0.521 vs. 0.52]; adding additional embeddings (such as age and/or county) to the baseline specification results in negligible differences in terms of ROC AUC. We also assess specifications with the patient’s zip code instead of the county given as additional embeddings and find that the model performance is below the base specification in terms of both APS and ROC AUC. In summary, our results suggest that, in the context of pediatric patients, augmenting a pre-trained model with information on the patient’s age at the time of medical encounter has a modest positive impact on model performance, while the addition of patient’s county of residence at the time of the visit does not improve the results further.

We proceed to evaluate the quality of our pre-trained embeddings through both intrinsic and extrinsic methods. Intrinsic assessment involves examining the embeddings’ quality through visual inspection and reporting cosine similarity among disease embeddings. For the extrinsic evaluation, we examine the embeddings’ effectiveness in predicting patient gender distribution for specific disease codes.

To visually inspect Ped-BERT’s embeddings, we reduce the embedding space to 2D using t-SNE (see scikit-learn^27^ for implementation details). Figure 4b shows the reduced embeddings for the baseline + age input embeddings specification. The visualization reveals that similar diseases (such as those related to injury and poisoning, diseases of the respiratory system, and birth conditions) cluster together. Furthermore, diseases known to frequently co-occur (such as neoplasms, diseases of the blood, and blood-forming organs) are also grouped closely. Upon closer examination of these 2D disease embedding clusters, a remarkable association with the International Classification of Disease Codes (ICD9 codes) becomes evident. Notably, this finding is interesting because we did not explicitly provide this information to Ped-BERT during the pre-training phase. Subsequently, we proceed to report the cosine similarity between disease codes using Ped-BERT’s learned embeddings. Upon aggregation at the chapter level, we observe a range of similarity values, with the minimum and maximum values being − 0.318 and 1, respectively; the values at the 25, 50, and 95 percentiles, are 0.093, 0.229, and 0.586, respectively (additional details are available in Supplementary Fig. S2).

Finally, we conduct an extrinsic evaluation of Ped-BERT’s embeddings by assessing their performance in predicting the gender distribution of patients with congenital anomalies and tuberculosis. This evaluation is prompted by the increasing body of evidence highlighting sex-specific disparities in the prevalence of congenital anomalies and tuberculosis, with research studies demonstrating higher prevalence rates among pediatric males^28,29^. As shown in Supplementary Fig. S3, Ped-BERT consistently predicts a higher prevalence of these two diseases among males when evaluated on the ‘pre-training validation set’, with a Fisher’s exact test value equal to 0.0862 (p $<0.1$).

In summary, our current intrinsic and extrinsic evaluation results indicate that Ped-BERT has developed a substantial understanding of the contextual relationships between diseases.

### Ped-BERT fine-tuning evaluation

The complete training procedure for Ped-BERT involves fine-tuning the model, which is initially trained as a general disease model for pediatric patients without task-specific objectives. In the fine-tuning stage, we adapt Ped-BERT to predict the principal medical diagnosis and the LoS in the subsequent pediatric visit. Specifically, we add a feedforward layer with 64 hidden units and an output layer with a softmax activation function on top of the pre-trained Ped-BERT. The model is fine-tuned for each task for 100 epochs using the Adam optimizer with a learning rate of $3e-4$ a dropout rate of 0.3, and early stopping.

To explore the impact of bidirectional self-attention versus constrained attention (where each disease token can solely attend to context on its left), we pre-train a transformer decoder (TDecoder) following the original transformer architecture^24^. This involves utilizing our ‘pre-training training set’ and ‘pre-training validation set’, similar to Ped-BERT, followed by fine-tuning the TDecoder model on our two downstream tasks. Additionally, we assess the efficacy of pre-training Ped-BERT compared to logistic regression (LR) and random forests (RF) classifiers, incorporating standard multi-hot inputs for up to three disease codes noted by clinical personnel during a medical encounter. We also introduce an untrained NN architecture (NN_REmb) with randomly initialized disease embeddings and a feedforward layer with 564 hidden units into our comparison framework.

For pre-training the TDecoder model, we use Ped-BERT’s hyperparameter configuration to facilitate comparative evaluation (refer to Supplementary Fig. S4 for pre-training APS and AUC results). The optimal architecture for LR, NN_REmb, and the fine-tuned TDecoder model involves training for 100 epochs using the Adam optimizer with a learning rate of $3e-4$ and early stopping. LR employs a dropout rate of 0.1, while NN_REmb and the fine-tuned TDecoder model utilize a dropout rate of 0.3. The optimal configuration for the RF model comprises 10 trees with a maximum depth of 5, coupled with balanced bootstrapped sampling.

Our experimental setup utilizing LR and RF algorithms focuses exclusively on the baseline embedding specification due to the course of dimensionality and memory limitations when age and county, as well as their respective interaction terms, are considered as features. Furthermore, given the inherent challenges decision tree models face in handling multiclass classification tasks with a large number of classes (in our case, 100+), we exclusively apply the RF model to the LoS prediction task. We report each model’s performance by taking the average of five independent runs.

**Figure 5:**
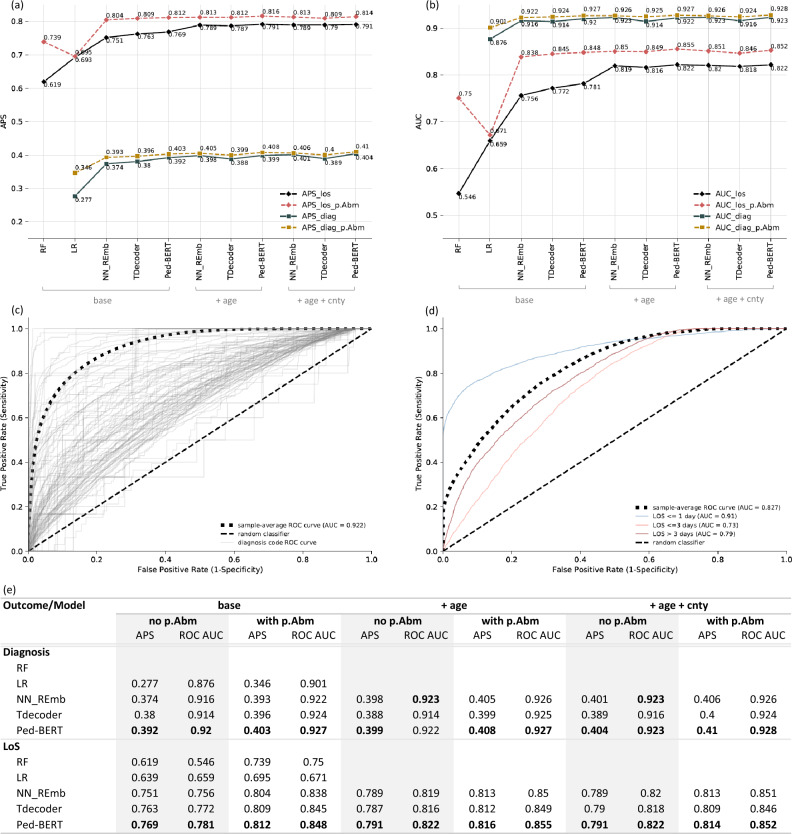
Evaluation of the disease and LoS prediction tasks. We consider the following input specifications: base embeddings, base + age embeddings, base + age + cnty embeddings, and multi-hot encoding. The embeddings are applied to the NN_REmb, TDecoder, and Ped-BERT models. For the LR and RF models we use the multi-hot inputs. For the NN_REmb model the embeddings are randomly initialized. For the TDecoder and Ped-BERT models, the embeddings are learned in the pre-training stage and fine-tuned in the fine-tuning stage. (**a**,**b**) The APS (left) and ROC AUC (right) computed for each model and input scenario outlined above (square-green and square-yellow lines represent the diagnosis prediction task, and diamond-black and diamond-red lines represent the LoS task; square-yellow lines and diamond-red lines augment each model with the mother’s attribute features in $p.A_{bm}$. The APS and ROC AUC metrics represent comparisons between the ground truth and the predicted diagnosis and associated LoS for each patient in the output partition of the input-output pairs of our ‘fine-tuning test set’. (**c**) True Positive Rates and False Positive Rates curves averaged across all patients for each diagnosis in the data (grey lines), and averaged across all patients for all diagnosis codes in the data (dot-dashed black lines); the long-dashed line denotes a random classifier. (**d**) similar to (**c**), but for the LoS prediction task, the colored lines representing performance for the three LoS classes. (**e**) similar to (**a**,**b**), but in tabular form for quick readability of results; the numbers in boldface indicate the highest score per category.

For the diagnosis prediction task utilizing base embeddings/multi-hot inputs, the DL models obtain the best results compared to the LR classifier (APS between 0.374–0.392 vs. 0.277, and ROC AUC between 0.914–0.92 vs. 0.876). Among the DL models, Ped-BERT stands out by significantly outperforming both the NN_REmb and TDecoder models in terms of both scores. The inclusion of additional embeddings such as age (+ age) or age + county (+ age + cnty) yields only marginal or negligible enhancements in ROC AUC across all models. However, there are improvements in APS, particularly for the NN_REmb model augmented with age embeddings, suggesting the potential utility of this additional feature for this architectural choice (Fig. 5a,b, square-green lines; Fig. 5e).

Similar trends, although with a larger magnitude, are observed for the LoS task with base embeddings/multi-hot inputs, whereby DL models outperform the LR and RF classifiers (APS between 0.751–0.769 vs. 0.619–0.693, and ROC AUC between 0.756–0.781 vs. 0.546–0.659). Once again, Ped-BERT outperforms both the NN_REmb and TDecoder models. Incorporating age embeddings into the base specification enhances the APS of the DL models by 2.2–3.8$\%$ and the ROC AUC by 4.1–6.3$\%$, effectively narrowing the performance gap between the DL models. No further improvements in performance are observed upon adding county embeddings to the base + age specification (Fig. 5a,b, diamond-black lines; Fig. 5e).

We next focus on diving deeper into Ped-BERT’s input configuration yielding the best prediction results. Specifically, in Fig. 5c, we report Ped-BERT’s ROC AUC for each principal diagnosis code as derived from the base + age embeddings specification. The results highlight Ped-BERT’s high predictive performance for specific conditions, including maternal causes of perinatal morbidity and mortality (AUC = 0.984), malignant neoplasm of genitourinary organs (AUC = 0.984), congenital anomalies (AUC = 0.945), pneumoconiosis and other lung diseases due to external agents (AUC = 0.903), and ischemic heart disease (AUC = 0.899). Conversely, lower prediction performance is observed for conditions such as hernia of abdominal cavity (AUC = 0.651), toxic effects of substances (AUC = 0.632), injury of nerves of spinal cord (AUC = 0.614), persons with potential health hazards related to personal and family history (AUC = 0.525), and injury to blood vessels (AUC = 0.496). Furthermore, Supplementary Fig. S5, assesses Ped-BERT’s suitability for detecting rare diseases, showing varying prediction performance levels across different diseases. Concretely, we report the ROC AUC scores for various genetic diseases listed as principal disease codes, including congenital anomalies of eyes (AUC = 0.912), cerebral degenerations manifesting in childhood (AUC = 0.677), diseases of white blood cells (AUC = 0.667), other diseases of the biliary tract (AUC = 0.660), diseases of the capillaries (AUC = 0.569), and other metabolic and immunity disorders (AUC = 0.560). For more details, refer to Supplementary Table S2, which provides additional information on the number of patients with these rare diseases in the ‘fine-tuning training set’, ‘fine-tuning validation set’ and ‘fine-tuning test set’. Finally, Fig. 5d illustrates Ped-BERT’s ROC AUC for the LoS prediction task based on the base+age specification. The prediction performance varies across different classes of LoS. Notably, the highest performance is observed for patients seen in an emergency or inpatient setting but discharged on the same day (LOS $\ge$ 1 day, AUC = 0.91). This is followed by the prediction of LoS in an inpatient setting for more than 3 days (LOS > 3 days, AUC = 0.79), and LoS in an inpatient setting lasting 2-3 days (LOS $\le$ 3 days, AUC = 0.73).

### The role of mother attributes data

To evaluate potential prediction enhancements for our two downstream tasks, and to further investigate the effectiveness of Ped-BERT’s pre-training, we expand our analysis by integrating the mother’s attributes data from the $p.A_{bm}$ set into all model configurations. We observe significant improvements.

In the diagnosis prediction task using base embeddings/multi-hot inputs, LR exhibits the most substantial gains with an APS and ROC AUC improvement of $6.9\%$ and $2.5\%$, respectively. The gains for the DL models are smaller or insignificant, with an APS ranging between 1–1.9$\%$ and a ROC AUC between 0.06–1$\%$. The DL models once again obtain the best results, with Ped-BERT slightly outperforming both the NN_REmb and TDecoder models in terms of APS score. Adding the mother’s health attributes data to the age (+ age) or age and county (+ age + cnty) embedding specification yields similar APS and AUC gains for the DL models (Fig. 5a,b, square-yellow lines; Fig. 5e).

Performance improvements are larger in magnitude for the LoS prediction task utilizing base inputs. The APS and ROC AUC gains for the non-DL models vary between 0.02–12$\%$ and 1.2–20.4$\%$, respectively. For the DL models, we observe APS and AUC improvements ranging from 4.3 to 5.3$\%$ and 6.7 to 8.2$\%$, respectively. Most importantly, incorporating pre- and post-partum mother health information significantly reduces the performance gap between the DL models. Finally, adding the mother’s data to the age (+ age) or age and county (+ age + cnty) embedding specification yields lower APS and AUC gains compared to the base specification (Fig. 5a,b, diamond-red lines; Fig. 5e). In Supplementary Fig. S6, we report Ped-BERT’s ROC AUC performance with these additional features, for each diagnosis code and LoS class as derived from the base + age embeddings specification.

### Fairness tasks

We are interested in determining whether next-visit diagnosis and LoS prediction errors are uniform across patient subgroups in our data. Figure 5 already gives us some insights into the APS and ROC AUC performance for these tasks (overall and by disease code or LoS class), but it is desirable to understand how well it performs for different subgroups. For example, Fig. 2 identifies groups of mother–baby/patient demographics and health-related outcomes belonging to the pairs used in this analysis. Our data also contains information on the mother’s country of birth, which is rarely available to research and unique to our study. As such, in this section, we aim to assess the fine-tuned Ped-BERT’s prediction performance with fairness in mind and use the pre-trained baseline + age embeddings specification for this task.

**Figure 6:**
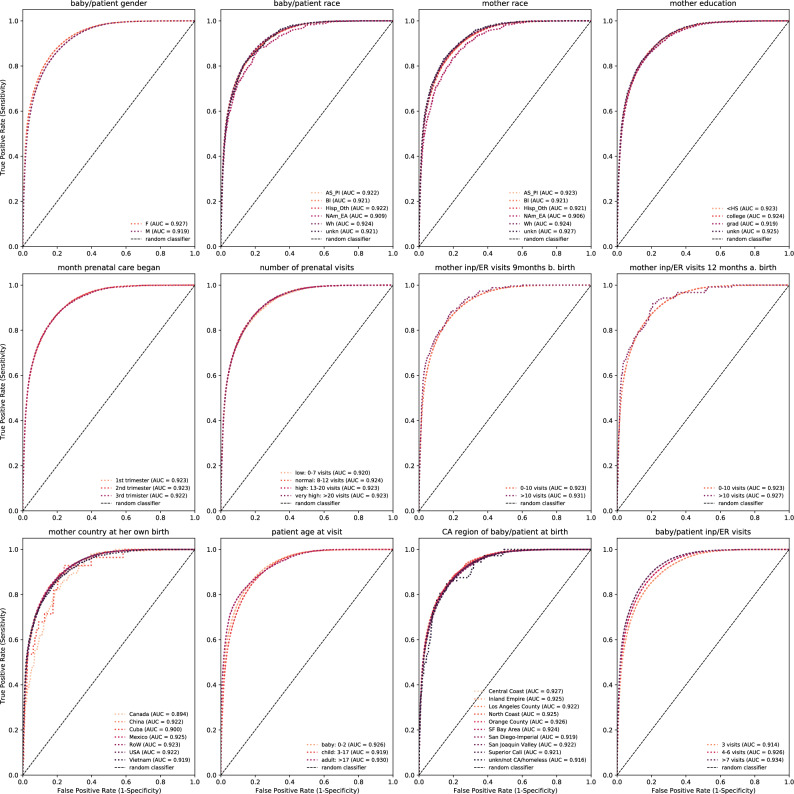
Fairness tasks for diagnosis prediction: We compare evaluation results across different patient subgroups (e.g., baby/patient gender and race; mother race and education; month prenatal care began, etc.). The evaluation results rely on the fine-tuned Ped-BERT model with base + age embeddings applied to the ‘fine-tuning test’ set. True Positive Rates (Sensitivity) vs. False Positive Rates (1 − Specificity) are shown as various shades of red-dotted lines. A long-dashed line denotes a random classifier.

For diagnosis prediction, we find minimal differences in ROC AUC performance across groups of patient gender and race, mother race and education, month prenatal care began, the number of prenatal visits, and the number of times the mother visited a healthcare facility overnight or in an ER setting (Fig. 6, top and middle panels). Next, we create bins for the mother’s country at her own birth, for similar patient ages, for zip codes/counties at birth belonging to the same geographical region^30^, and for the number of times a patient was seen in an inpatient/ER setting. We find that Ped-BERT is slightly more susceptible to prediction errors depending on mother’s country of origin at her own birth and for patients with shorter medical histories (Fig. 6, bottom panel). Additionally, the integration of the mother’s health attributes data yields enhancements in ROC AUC performance across all demographic subgroups within our dataset, rather than solely in the overall evaluation (refer to Supplementary Fig. S7).

**Figure 7:**
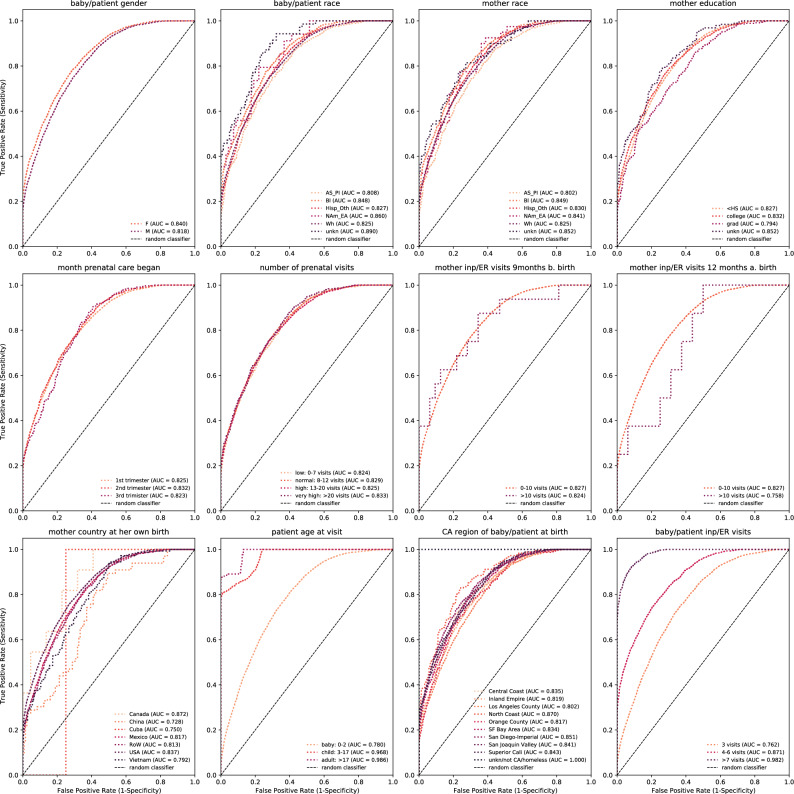
Fairness tasks for LoS prediction: Similar to Fig. 6 but for the LoS prediction task.

In contrast, we find significant subgroup differences in ROC AUC performance for the LoS prediction task. For example, Ped-BERT has better LoS performance for females relative to males (AUC 0.840 vs. 0.818), for patients whose mothers had less than 10 inpatient/ER visits in the post-partum period as opposed to more than 10 (AUC 0.827 vs. 0.758), for patients aged 3 years and above (AUC 0.780 for 0–2 years old vs. 0.968 for 3–17 years old and 0.986 for 17+ years old), and for patients with a longer history of medical visits (AUC 0.762 for 3 visits vs. 0.982 for more than 7 visits and 0.871 for 4–6 visits (Fig. 7). Once again, the integration of the mother’s attributes data in $p.A_{bm}$, leads to ROC AUC performance improvements across all subgroups in our data (refer to Supplementary Fig. S8).

### Research application

Our study allows medical researchers to assess optimal machine learning model configurations for enhancing diagnosis accuracy and LoS predictions based on available input features. For example, Ped-BERT could be a good architectural choice when age information is generalized or masked to protect patient identities due to privacy regulations like the Health Insurance Portability and Accountability Act (HIPAA). These research outcomes, in turn, hold potential significance for clinical practitioners, as they could integrate machine learning insights into their decision-making processes, thereby addressing uncertainties associated with potential medical conditions. This integration facilitates informed scheduling of follow-up appointments, optimizing patient care delivery and potentially diminishing anticipated LoS.

## Discussion

This research aims to improve the early detection of diseases and associated LoS for pediatric patients by leveraging a unique healthcare database and the latest developments in bidirectional encoder representations from transformers (BERT). The fine-tuning data used in our analysis consists of vital statistics and birth information, as well as hospital discharge data and emergency room visits in California between 1991 and 2017 for 513,963 mother-baby pairs. A BERT-based model called Ped-BERT, pre-trained using an even larger corpus of data and a masked language model (MLM) approach, is able to accurately predict the likelihood of over 100 conditions and associated LoS in a child’s subsequent medical visit. This study also assesses Ped-BERT’s prediction performance gains when incorporating mother’s attribute data (such as pre- and post-partum health history), and evaluates fairness by assessing whether prediction errors are uniformly distributed across different mother-baby demographics and health characteristics subgroups. The findings suggest that Ped-BERT holds promise in aiding researchers in leveraging machine learning for pediatric healthcare recommendations and supporting clinical decision-making processes.

The pre-training stage of Ped-BERT involves learning good representations of diseases by testing different combinations of input embeddings to represent a patient’s health history. The base specification is the sum of diagnosis embeddings and positional encodings. Age and zip/county embeddings augment this baseline in our performance improvement tests. We find that adding age embeddings improves the APS and ROC AUC score relative to baseline, and further expanding with county embeddings results in negligible performance differences relative to the base + age specification. We use intrinsic and extrinsic methods to evaluate the embedding quality further. Intrinsically, we find that the model has learned to cluster together diseases that belong to the same ICD9 chapter or are known to co-occur. Extrinsically, we find that the disease embeddings generated by Ped-BERT correctly predict the male-skewed gender distributions for congenital anomalies and tuberculosis.

The fine-tuning stage of the Ped-BERT model involves adapting the model for our specific downstream tasks. To investigate the impact of bidirectional self-attention compared to left-to-right attention, we also pre-train and fine-tune a transformer decoder model (TDecoder). Furthermore, we evaluate the effectiveness of pre-training Ped-BERT in comparison to logistic regression and random forests classifiers, as well as an untrained neural network architecture featuring randomly initialized disease embeddings.

Our findings reveal the superiority of DL models over non-DL models in both prediction tasks. The fine-tuned Ped-BERT model with base embeddings outperforms other DL models for both prediction tasks, and adding age to the base embedding specification leads to similar performance across the DL models. Expanding our fine-tuning analysis by integrating the mother’s health data leads to significant APS and ROC AUC improvements across all models, overall, and for each subgroup in our data, particularly for the LoS task. Finally, we assess the fine-tuned Ped-BERT for fairness, as models that perform poorly on certain subgroups can lead to unequal outcomes and perpetuate biases. Ped-BERT generally performs similarly across all subgroups in our analysis for the diagnosis prediction task. However, larger discrepancies are observed for the LoS task.

We propose several possible directions for future research based on the architecture and properties of Ped-BERT. For example, one can focus on pre-training and fine-tuning the model for early detection of rare genetic pediatric conditions or for predicting hospital LoS for specific diseases. Another possibility is improving the prediction performance for the LoS task by incorporating hospital zip/county information and patient insurance type as features. This information is available in the HCAI^20^ dataset and would require additional data cleaning.

### Supplementary Information

Supplementary Information.

## Data Availability

We collect health data from The California Department of Health Care Access and Information (HCAI^20^), which provides confidential patient-level data sets to researchers eligible through the Information Practices Act (CA Civil Code Section 1798 et seq.). Note that researchers interested in working with this health data should request it directly from HCAI (https://hcai.ca.gov/data-and-reports/research-data-request-information/) as it is HIPAA protected, and by agreement, we are not allowed to distribute it. The geospatial data comes from the Census Bureau and includes 2010 ZCTA shapefiles (https://www.census.gov/cgi-bin/geo/shapefiles/index.php?year=2010 &layergroup=ZIP+Code+Tabulation+Areas_)^31^, 2010 county shapefiles (https://www.census.gov/cgi-bin/geo/shapefiles/index.php?year=2010 &layergroup=Counties+%28and+equivalent%29)^32^, 2010 ZCTA to county codes (https://www.census.gov/programs-surveys/geography/technical-documentation/records-layout/2010-zcta-record-layout.html)^33^, ZCTA to zip codes crosswalks (https://github.com/censusreporter/acs-aggregate/blob/master/crosswalks/zip_to_zcta/ZIP_ZCTA_README.md)^34^, as well as the 2020 geographical division of California’s 58 counties into ten regions (https://census.ca.gov/regions/)^30^. The underlying code for this study, including data preprocessing and analysis, is publicly available at https://github.com/corneliailin/CA_hospitals_online.
