# Supplementary material for: Early detection of pediatric health risks using maternal and child health data
Source: Sci Rep. 2024 Jul 4;14:15350. doi: 10.1038/s41598-024-65449-8 (PMC11222373; doi:10.1038/s41598-024-65449-8)
Supplement: Supplementary file 1 — Supplementary Information. [file 41598_2024_65449_MOESM1_ESM.pdf]

Appendices

A Data Acquisition and Processing

A.1 Health Data

Our data is distributed by the California Department of Health Care Access and Information (HCAI[20]). We requested the following files for research purposes:

Linked Birth Files (Birth data): a research database created to study delivery and birth outcomes. It includes maternal antepartum and postpartum hospital records for the nine months before delivery and one-year post-delivery. In addition, the linked file contains birth records and all infant readmissions occurring within the first year of life. The file contains all infants born in a given year, including births that happened in a California hospital that reported to HCAI, births that occurred in a California hospital that did not report to HCAI, and births that occurred outside California. It includes all infants and mothers, irrespective of whether they were linked to a birth record. The linked pairs of birth/delivery records have information associated with a mother/baby pair from the baby's discharge data record, the mother's discharge data record, and the birth certificate data. Linked birth files are available beginning with the 1991 calendar year reporting period (HCAI[20]).

The Patient Discharge Dataset (PDD): consists of a record for each inpatient discharge from a California-licensed hospital. Licensed hospitals include general acute care, acute psychiatric, chemical dependency recovery, and psychiatric health facilities. These datasets are available starting in 1983 (HCAI[20]). For more information on the data and reporting requirements, see the California Inpatient Data Reporting Manual.[35]

The Emergency Department Dataset (EDD): includes information from hospitals licensed to provide emergency medical services. The EDD encounters include those patients who had face-to-face contact with the provider. If the patient left without being seen, the patient would not have had a face-to-face encounter with a provider, and therefore the EDD encounter would not be reported. These data sets are available beginning January 2005 (HCAI[20]).

Our study's primary variable of interest is the primary, secondary, and tertiary ICD 9 or ICD10 diagnosis codes at the time of visits, as well as the hospital length of stay determined at discharge time. We access this data along with other relevant metadata, such as mother-baby demographics and mother health-related outcomes nine months before and 12 months after birth.

A.2 Geospatial Data

The geospatial data is constructed and made available by the Census Bureau. For California, the relevant 2010 ZCTA and county-specific shapefiles,[31,32] the 2010 ZCTA to county codes,[33] and the ZCTA to ZIP crosswalks[34] are identified and mapped to our health data for visualization and analysis purposes. For the Fairness analysis, we extract the California - Census 2020 geographical division of counties into regions. Supplementary Table S0 contains a summary of the counties used for each region.

Supplementary Table S0: **Geographical division of California's counties** - Details of counties included in each California region to support the Fairness analysis presented in Figure 6-7 and Supplementary Figure 7-8

Region	County
Central Coast	Monterey, San Benito, San Luis Obispo, Santa Barbara, Santa Cruz, Ventura
Inland Empire	Riverside, San Bernardino
Los Angeles County	Los Angeles
North Coast	Del Norte, Humboldt, Lake, Mendocino, Napa, Sonoma, Trinity
Orange County	Orange
SF Bay Area	Alameda, Contra Costa, Marin, San Francisco, San Mateo, Santa Clara, Solano
San Diego - Imperial	Imperial, San Diego
San Joaquin Valley	Alpine, Amador, Calaveras, Madera, Mariposa, Merced, Mono, San Joaquin, Stanislaus, Tuolumne, Fresno, Inyo, Kern, Kings, Tulare
Superior Cali	Butte, Colusa, El Dorado, Glenn, Lassen, Modoc, Nevada, Placer, Plumas, Sacramento, Shasta, Sierra, Siskiyou, Sutter, Tehama, Yolo, Yuba

.

B Supplementary Tables

Experiment	Model name (if existing paper)	Layers (L)	Hidden Size (H)	Self-attention Heads (A)	FF size	APS	AUC
1	Med-BERT	6	192	6	64	0.47	0.91
2	BEHRT	6	288	12	512	0.49	0.93
**3**	**Ped-BERT_v1**	**6**	**128**	**12**	**128**	**0.51**	**0.95**
4	Ped-BERT_v2	6	128	12	512	0.5	0.95

Supplementary Table S1: **Ped-BERT hyperparameter search.** Ped-BERT is tuned using the baseline embeddings specification (i.e., the sum of diagnosis embeddings and positional encoding) and by comparing the APS and ROC AUC results of the 'pre-training train set' and 'pre-training validation set'. The optimal architecture, which we present in the main text of the paper, is highlighted here with maroon (i.e., Ped-BERT_v1).

two-digit ICD9 diag code	# of patients in training	# of patients in validation	# of patients in test
27	4115	394	1054
28	1381	131	307
33	496	51	117
44	201	17	61
57	894	86	220

Supplementary Table S2: **Rare Genetic Diseases Prediction Task.** Number of patients in the 'fine-tuning training set', 'fine-tuning validation set', and 'fine-tuning test set' for selected rare genetic diseases specific to pediatric patients, at the two-digit ICD9 code level.

C Supplementary Figures

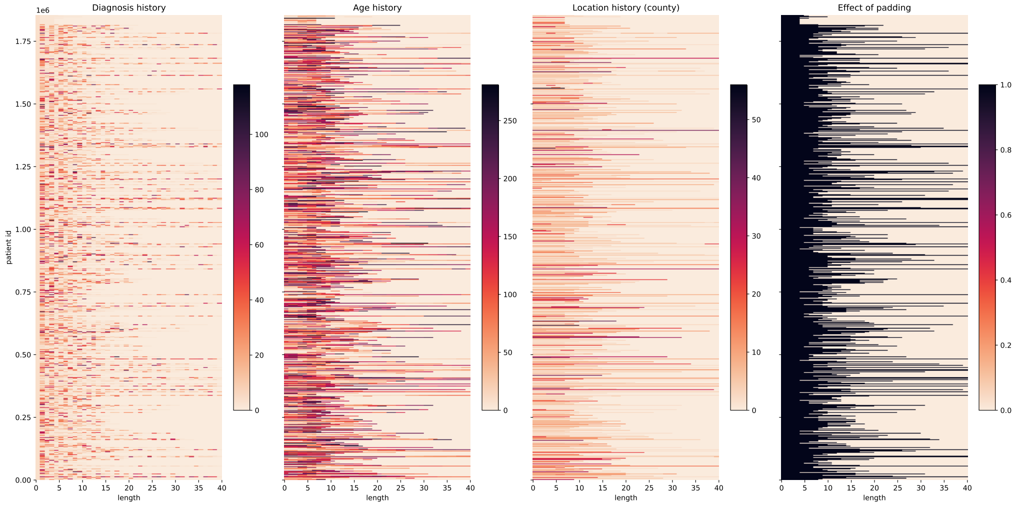

Supplementary Figure S1: **Summary statistics of encoded input for pre-training Ped-BERT**. The x-axis represents the length of a given patient history, which we optimally set to 40 periods. Each tick on the y-axis represents a diagnosis, age, location history, and padding summary for a given patient ID in the pre-training data. Heatmap values and colors represent: for **(a-c)**, the encoded disease codes, age, and location history; for **(b)**: the effect of zero padding since not all patients have a history length equal to 40.

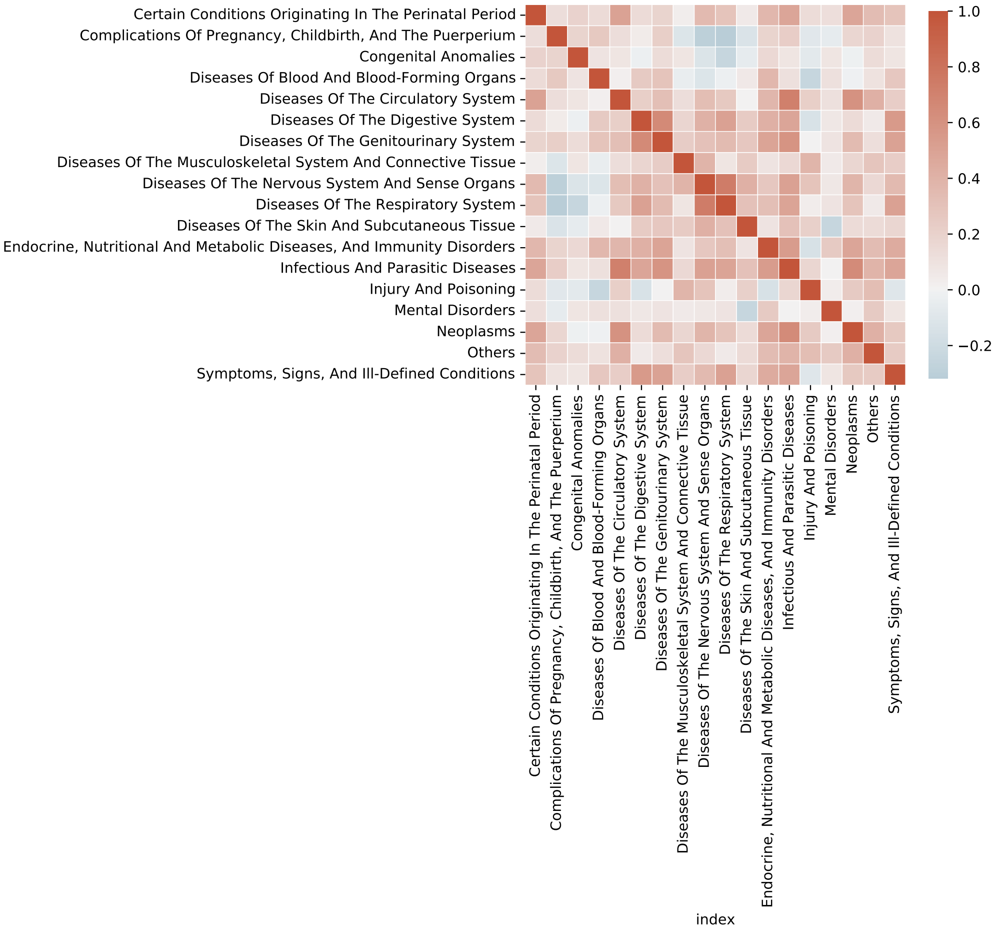

Supplementary Figure S2: **Intrinsic evaluation of embeddings**. Learned embeddings are extracted from the pre-training stage for the base + age input embedding specification. The heatmap represents the cosine similarity for all the diagnosis codes in our data aggregated at the chapter level. Negative values (blue shades) reflect opposite similarities, and positive values (red shades) represent close similarities.

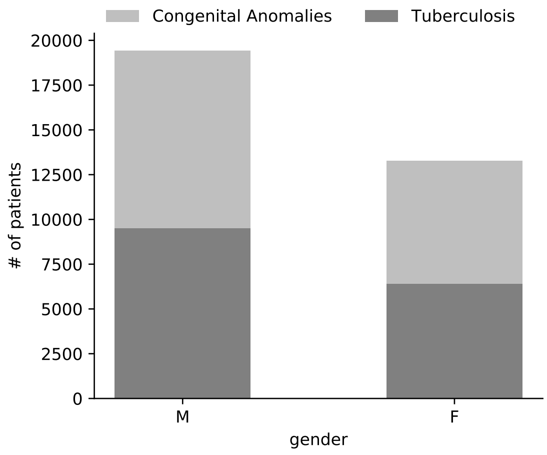

Supplementary Figure S3: **Extrinsic evaluation of embeddings**. Assess the performance of the pre-trained Ped-BERT model in predicting the patient gender distribution for congenital anomalies (light gray) and tuberculosis (dark gray). The y-axis represents the number of patients predicted to have the given diseases. The results presented here rely on the base + age embeddings specification using the 'pre-training validation set'. Abbreviations: F = Female, M = Male.

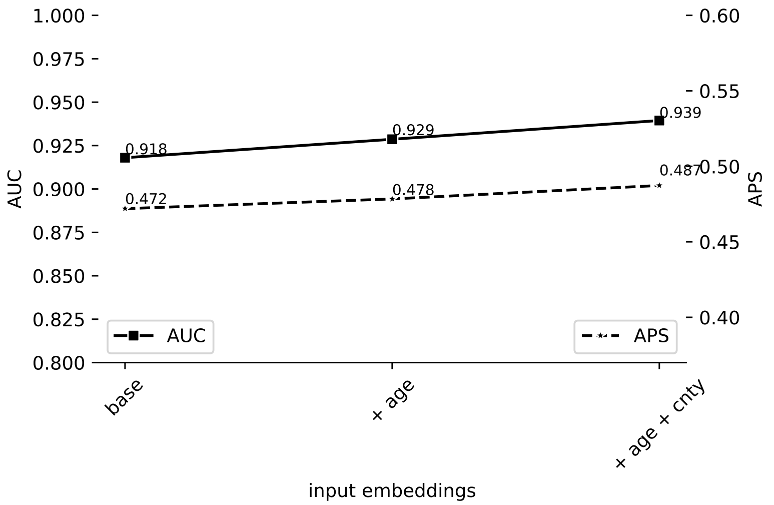

Supplementary Figure S4: **Evaluation of the TDecoder pre-training task**. The average precision score (APS, right y-axis) and the area under the receiver operating curve (ROC AUC, left y-axis) are computed as sample averages for the following embedding specifications: base, base + age, and base + age + county embeddings. These metrics represent comparisons between the ground truth (primary diagnosis token at the next medical encounter) and the TDecoder-predicted primary diagnosis token.

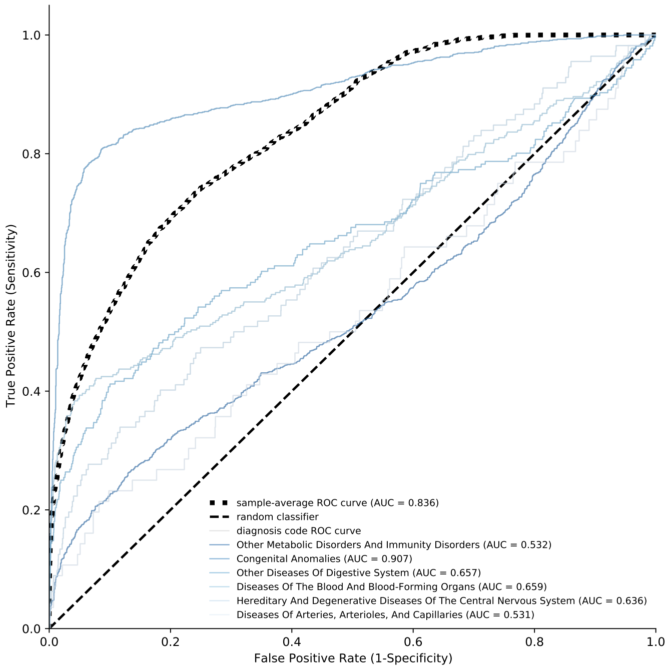

Supplementary Figure S5: **Evaluation of the Disease Prediction Task**. Similar to Figure 5c, but for rare genetic diseases only.

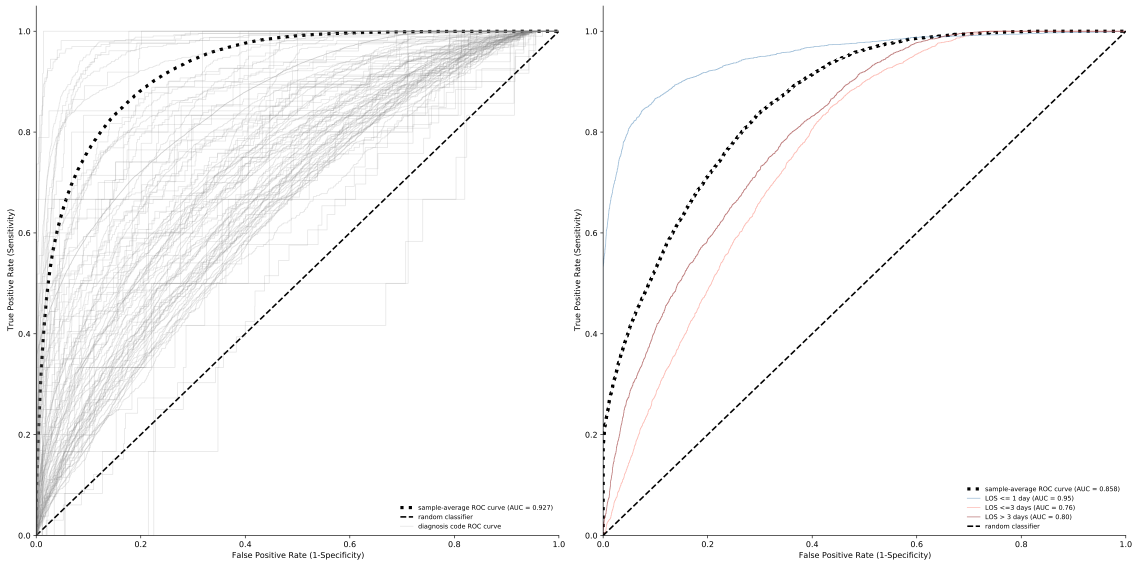

Supplementary Figure S6: **Evaluation of the Disease Prediction and LoS Tasks**. Similar to Figure 5(c-d), but the features are now augmented with the mother's attributes data in *p.Abm*.

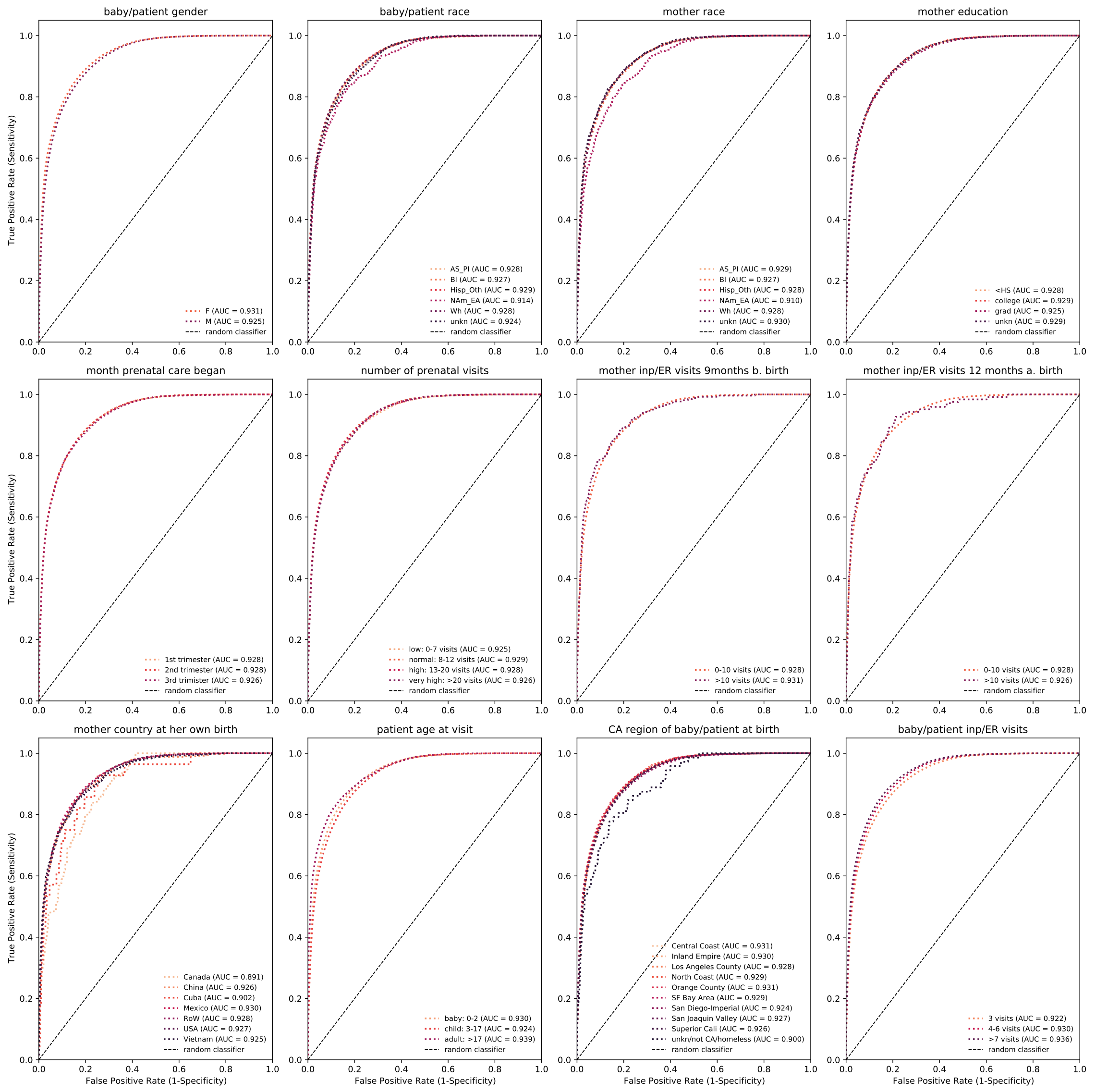

Supplementary Figure S7: **Fairness Tasks for Diagnosis Prediction**. Similar to Figure 6, but the features are now augmented with the mother's attributes data in $p.A_{bm}$.

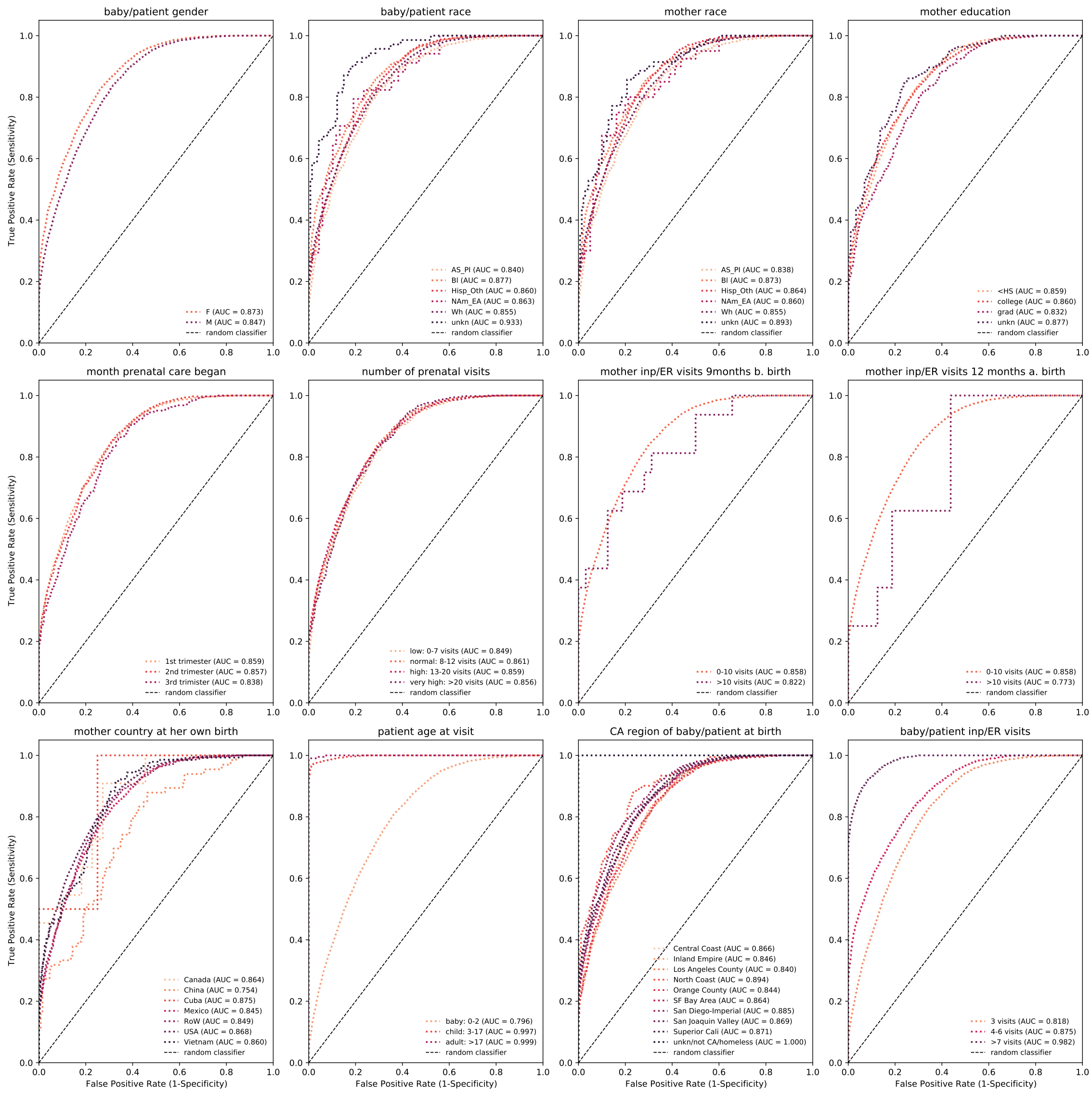

Supplementary Figure S8: **Fairness Tasks for LoS Prediction**. Similar to Figure 7, but the features are now augmented with the mother's attributes data in $p.A_{bm}$.
